# TIGIT enhances CD4^+^ regulatory T‐cell response and mediates immune suppression in a murine ovarian cancer model

**DOI:** 10.1002/cam4.2976

**Published:** 2020-03-25

**Authors:** Fengzhen Chen, Yanying Xu, Yulong Chen, Shu Shan

**Affiliations:** ^1^ Department of Gynecology The Second Hospital of Tianjin Medical University Tianjin China; ^2^ Department of Lung Oncology Tianjin Medical University Cancer Institute and Hospital Tianjin China; ^3^ Department of Gynecology and Obstetrics Affiliated Tongji Hospital Tongji University Shanghai China

**Keywords:** immunosuppression, ovarian cancer, regulatory T cell, TIGIT

## Abstract

Ovarian cancer (OC) is the fifth‐leading cause of cancer‐related death in women with a pathogenesis involving activation of regulatory T cells (Tregs). The T‐cell immunoglobulin and ITIM domain (TIGIT) is a well‐known immune checkpoint molecule that inhibits T‐cell responses. However, the role of TIGIT in OC is not comprehensively understood. In this study, we revealed crucial functions of TIGIT in the development and progression of OC. ID8 cells were used to establish a murine OC model. TIGIT expression was increased in immune cells of OC mice, particularly in CD4^+^ Tregs. Anti‐TIGIT monoclonal antibodies (mAb) were used to block the function of TIGIT in OC mice, and we found that the anti‐TIGIT treatment reduced the proportion of CD4^+^ Tregs, but did not affect CD4^+^ and CD8^+^ T cells or natural killer cells. Splenic CD4^+^ Tregs from OC mice were isolated after the anti‐TIGIT treatment, and their functioning was examined. Inhibition of TIGIT lowered the degree of immunosuppression induced by CD4^+^ Tregs. A survival curve suggested that anti‐TIGIT treatment can improve the survival rate of OC in mice. We conclude that TIGIT enhanced CD4^+^ Tregs response and mediated immunosuppression in the OC model. Our data suggest that inhibition of TIGIT is a potential therapeutic target in OC patients.

## Graphical abstract

TIGIT enhanced CD4^+^ Tregs response and mediated immune suppression in OC; TIGIT is a potential target in OC patients.

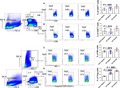

## 1 INTRODUCTION

Ovarian cancer (OC), the fifth‐leading cause of cancer‐related death in women, was diagnosed in 22 240 patients and led to an estimated 14 070 deaths in the United States in 2018.^1^ This high lethality of OC is primarily due to limited infrequency of screening and unspecific symptoms, resulting in diagnosis only at an advanced stage (International Federation of Gynecology and Obstetrics stage III and IV). Early‐stage OC can be cured through surgery and chemotherapy in up to 90% of the cases; however, after progression to advanced OC stages, the cure rate decreases to less than 30%.^2^ The standard and first‐line treatment of OC is surgical debulking followed by platinum‐taxane‐based chemotherapy.^3^ Although approximately 80% of OC patients initially respond to chemotherapy, more than 60% suffer a relapse with fatal outcome.^4^ Therefore, novel complementary methods are urgently needed to increase OC therapy success.

OC includes various histological and genetic forms of tumors from epithelial, germ cell, or sex cord‐stromal origin. Some tumors primarily arise from tissues which are not present in the ovary under normal conditions.^5^ Infiltration of more effector T cells and fewer regulatory T cells (Tregs) into OCs was shown to be strongly associated with better chances of survival.^6^ T cells are activated by co‐regulatory signals to enhance antitumor immune responses when tumor antigens occur. Negative checkpoints such as PD‐1/PD‐L1 and CTLA‐4 can also be activated during OC and subsequently suppress antitumor immune responses. Increased expression of such negative checkpoints during OC typically indicate a worse prognosis.^7^ Several studies confirmed that blocking of negative checkpoints may result in substantial clinical benefit^8^, ^9^, ^10^, ^11^; this approach thus opens new avenues for immunotherapy of OC.

The T‐cell immunoglobulin and ITIM domain (TIGIT) is a member of the immunoglobulin superfamily. It is expressed exclusively on lymphocytes and particularly on effector and regulatory CD4^+^ T cells, effector CD8^+^ T cells, and natural killer (NK) cells.^12^ Like CTLA‐4 and PD‐1, TIGIT is a negative checkpoint of the tumor immune response. Under competitive interaction with CD226 and CD96 for binding to CD155, TIGIT may disrupt CD226 co‐stimulation and produce inhibitory signals leading to suppression of antitumor immune responses.^13^ A recent study indicated that TIGIT predominantly regulates immune responses through CD4^+^ Tregs,^14^ whereas CD4^+^ Tregs abundance was correlated with tumor burden in OC patients.^15^ Therefore, we hypothesized that TIGIT‐mediated immune suppression through enhancing CD4^+^ Tregs responses during OC. In this study, we found evidence of TIGIT to increase CD4^+^ Tregs responses, and blocking of TIGIT exerted therapeutic effects in an OC model.

## 2 METHODS

### 2.1 Study animals

Female C57BL/6 mice (6‐8 weeks old) were purchased from the Laboratory Animal Center of the Chinese Academy of Medical Sciences and were housed in ventilated cabinets under standard conditions (temperature 21 ± 2°C, 50%‐60% relative humidity, and a 12/12 hours light/dark cycle). All animal experiments were approved by the ethics committee of the Tianjin Medical University. Mice (8‐10 individuals per group) received an intraperitoneal (i.p.) injection with 1 × 10^6^ ID8 cells ten days before the treatments. OC mice were injected thrice in 4‐day intervals with 100 μg of either control medium (Ultra‐LEAF™ Purified Mouse IgG1, κ Isotype Ctrl Antibody, Catalog #401414, BioLegend, San Diego, CA) or with anti‐TIGIT mAb (absolute IgG1 antibody, clone 1B4, Catalog #Ab01258, BioLegend, San Diego, CA).^16^ After 7 days, the spleen and ascites were collected from treated mice, and mononuclear cells were isolated and analyzed using flow cytometry. For survival experiments, mice (20 individuals per treatment group) were injected i.p. with 1 × 10^6^ ID8 cells 10 days before the treatment. The treatment groups were injected thrice with 100 μg of control medium or anti‐TIGIT mAb in 4‐day intervals. Mice were weighed twice per week and were examined daily for signs of ascites formation such as a swollen abdomen. Signs of toxic effects such as weight loss, hunched posture, mobility, diarrhea, anorexia, and respiratory problems were monitored on a daily basis. Mice were killed according to institutional guidelines when they developed ascites and showed weight increase of >30%. A Kaplan‐Meier survival curve and log‐rank tests were used to calculate mean survival times.

### 2.2 Cell lines

ID8 is a commonly known clone of the MOSEC ovarian carcinoma of C57BL/6 origin (Catalog #SCC145), which was supplied by the University of Pennsylvania. Before preparing cell suspensions and administering them to mice, ID8 cells were cultured at 37°C and 5% CO_2_ in complete DMEM medium supplemented with 10% FBS (Catalog #10099, Gibco), 100 U/mL penicillin, and 100 μg/mL streptomycin.

### 2.3 Antibodies and flow cytometry analysis

Flow cytometry analysis was performed up to three times to characterize immune cell phenotypes in the spleen and ascites. Immune cells were stained using the following mAbs: CD3 PerCP (Catalog #145‐2C11, BD Biosciences), CD4 Pacific Orange (Catalog #MCD0430, Invitrogen), CD8 Pacific Blue (Catalog #344718, BioLegend), CD25 PE‐Cy7 (Catalog # 552880, BD Biosciences), NK‐1.1 FITC (Catalog #553164, BD Biosciences), FoxP3 PE (Catalog #560408, BD Biosciences), and TIGIT BV605 (Catalog #744212, BD Biosciences). Mononuclear cells (1.5 × 10^6^ cells) isolated from the spleen and ascites were incubated with antibody mixtures prepared using FACS buffer (PBS containing 2% BSA and 0.05% sodium azide) at 1:100 for 15 minutes at 4°C and were then washed twice. Cells were fixed and permeabilized using a BD Cytofix/Cytoperm kit (Catalog #554714, BD Biosciences) to perform intracellular staining according to the manufacturer's instructions. After staining, cells were immediately applied to a BD LSR II multicolor flow cytometer (BD Biosciences), and data were analyzed using FlowJo software version 10 (Tree Star).

### 2.4 Isolation of splenic CD4^+^CD25^+^ TREGS and CD4^+^CD25^−^ T effector cells

Spleens were harvested and prepared as single‐cell suspensions by passing through 40‐μm filters twice, and mononuclear cells were then isolated by the Ficoll‐Paque density gradient centrifugation. CD4^+^CD25^+^ Tregs and CD4^+^CD25^−^ T effector cells were isolated using a commercially available mouse CD4^+^CD25^+^ Tregs isolation kit (Catalog #130‐091‐041, Miltenyi Biotec).^17^ Isolated cells were cultured in RPMI 1640 supplemented with 10% FBS.

### 2.5 Co‐culture

Splenic CD4^+^ Tregs were isolated from normal mice and OC mice that had received no treatment, from isotype controls, and from anti‐TIGIT‐treated mice. Cells were then co‐cultured with normal splenic CD4^+^CD25^−^ T effector cells from normal mice for 24 hours in a ratio of 1:1 (2 × 10^5^ cells per well in total) and were subsequently treated with anti‐CD3 (5 μg/mL) and anti‐CD28 (2 μg/mL) for polyclonal activation of T cells. We used CCK‐8 and Annexin‐V staining to examine proliferation and apoptotic rates in CD4^+^CD25^−^ T effector cells, and we used enzyme‐linked immunosorbent assays (ELISA) to assess secretive ability (IFN‐γ and IL‐4) of CD4^+^CD25^−^ T effector cells.

### 2.6 CCK‐8 measurement

After co‐culture for 24 hours, cells in the supernatant were collected and seeded on 96‐well plates at a density of 1 × 10^5^ cells per well using three replicates. Then, 10 μL CCK‐8 (Dojindo Molecular Technologies, Inc) was added to each well, and cells were incubated for 4 hours at 37°C without light exposure. Absorbance was measured using a microplate reader (Spectra MR, Dynex) at OD 450 nm.

### 2.7 Annexin‐V staining

An annexin V‐fluorescein isothiocyanate apoptosis kit (Nanjing Keygen Biotech) was used to measure cell apoptosis, according to the manufacturer's instructions. Briefly, cells were resuspended in 100‐μL binding buffer containing 5 μL Annexin‐V and 5 μL 7‐AAD and were incubated for 15 minutes at room temperature. Cells were then washed twice using cold PBS and were subsequently resuspended in a 300‐μL binding buffer. Flow cytometry was performed within 1 hour. Percentages of Annexin‐V‐positive cells are shown in the results section.

### 2.8 ELISA

Co‐culture supernatants were collected to measure IFN‐γ and IL‐4 levels using ELISA kits (Catalog #BMS606 and #BMS613, Invitrogen). The procedure was performed strictly following the manufacturer's instructions. Briefly, the supernatant was diluted at a ratio of 1:1 using the sample dilution buffer, and 100 µL of the mixture was placed in each well. To produce a calibration curve, a dilution series of the standard was applied to the same plate, which was then incubated at 37°C for 1 hour. The liquid was then removed, and the plate was washed five times using a washing solution. An enzyme‐labeled secondary antibody was diluted using a sample dilution buffer, and 100 µL was added to each well, followed by incubation at 37°C for 1 hour. The secondary antibody was then removed, and the plate was again washed five times using washing solution. A substrate solution was then added to the wells, and the reaction was incubated. When sufficient coloration was reached, the reaction was stopped by adding stop solution, and absorption was measured at 450 nm using a plate reader (Spectra MR, Dynex).

### 2.9 Statistical analyses

A Kaplan‐Meier survival curve was produced, and group effects on survival rates were tested using a log‐rank test. One‐way analysis of variance followed by the Bonferroni's test was used for all other assays. All results were derived from three independent experiments. Statistical analyses were conducted using GraphPad Prism Version 5. An alpha value of *P* < .05 was considered statistically significant.

## 3 RESULTS

### 3.1 TIGIT was higher expressed in OV mice than in naïve mice

**Figure 1:**
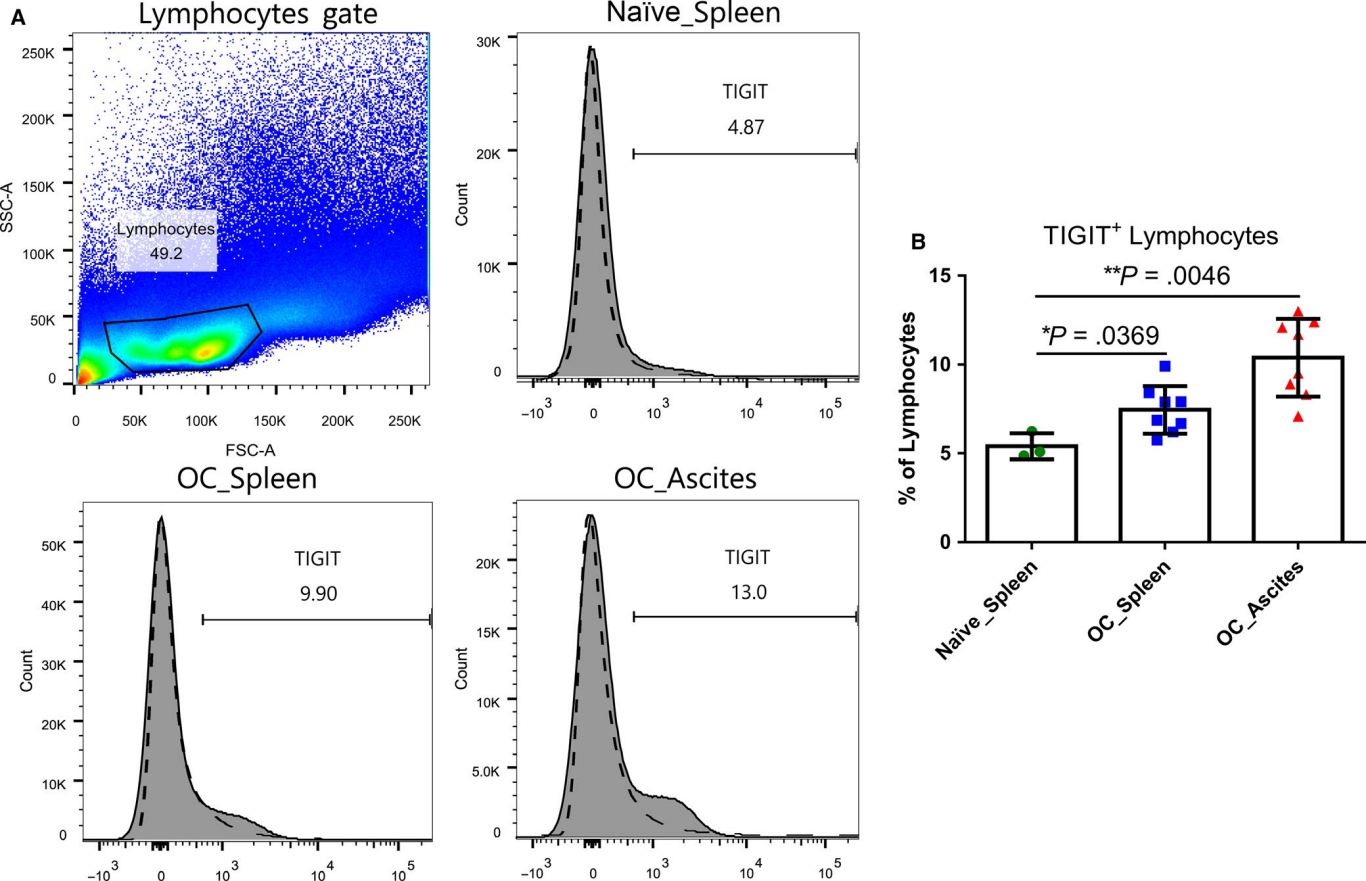
Expression of TIGIT on lymphocytes of a murine OC. Mice (n = 8) were injected i.p. with 1 × 10^6^ ID8 cells. Seven days after injection, peritoneal lavage fluid and the spleen of OC and normal mice (n = 3) were collected, and mononuclear cells were isolated by Ficoll‐Paque density gradient centrifugation. The proportion of TIGIT + lymphocytes was assessed using flow cytometry (A). Dashed lines indicate TIGIT isotype controls; the shaded are in the histogram indicates TIGIT expression (B). “Naïve” indicates normal murine. Shown are the means ± standard deviation

To assess the mechanisms underlying immune dysregulation during OC, wild‐type mice were injected with ID8 cells to establish the OC model and were killed 7 days after tumor inoculation. Spleens and ascites were harvested, and mononuclear cells were collected by Ficoll‐Paque density gradient centrifugation. TIGIT on lymphocytes was assessed using flow cytometry. We found that in OC mice, TIGIT was highly expressed than in naïve individuals, on lymphocytes of the spleen (7.45 ± 0.48% vs 5.40 ± 0.42%, *P* = .0369) and ascites (10.38 ± 0.77% vs 5.40 ± 0.42%, *P* = .0046; Figure 1).

### 3.2 Elevated TIGIT was expressed predominantly on CD4^+^ TREGS, compared to CD4^+^, CD8^+^ T cells, and NK cells

**Figure 2:**
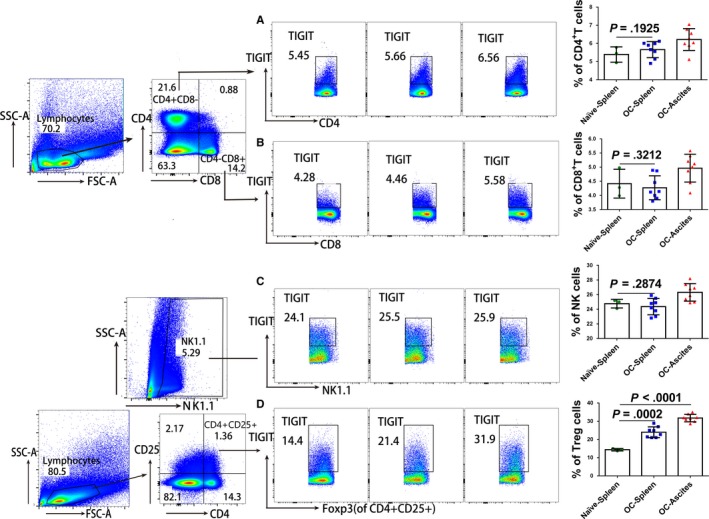
TIGIT expression on distinct immune cell types in OC mice. Mice were injected i.p. with 1 × 106 ID8 cells. Seven days after injection, peritoneal lavage fluid and the spleen of OC mice (n = 8) and normal individuals (n = 3) was stained and was examined for the proportion of TIGIT + for CD4^+^ T cells (A), CD8^+^ T cells (B), NK cells (C), and CD4^+^ Tregs cells (D) using flow cytometry. Shown are the means ± standard deviation

To determine expression of TIGIT on distinct immune cell types in OC mice, mononuclear cells of spleen and ascites were collected by Ficoll‐Paque density gradient centrifugation 7 days after establishing the model and were stained and analyzed using flow cytometry. We used NK1.1^+^ to determine NK cells and CD4^+^CD25^+^Foxp3^+^ to determine CD4^+^ Tregs. No difference in TIGIT expression was observed between OC mice and naïve individuals in NK cells (24.36 ± 0.40% vs 24.77 ± 0.34%, *P* = .2874), CD4^+^ T cells (5.66 ± 0.16% vs 5.39 ± 0.24%, *P* = .1925), and CD8^+^ T cells (4.27 ± 0.15% vs 4.42 ± 0.29%, *P* = .3212). However, CD4^+^ Tregs in the spleen (23.98 ± 1.05% vs 14.37 ± 0.38%, *P* = .0002) and in ascites (31.74 ± 0.73% vs 14.37 ± 0.38%, *P* < .0001) of OC mice showed substantially higher TIGIT expression levels than in those of naïve mice (Figure 2).

### 3.3 Anti‐TIGIT treatment reduced the proportion of CD4^+^ TREGS but did not affect CD4^+ ^and CD8^+^ T cells and NK cells

**Figure 3:**
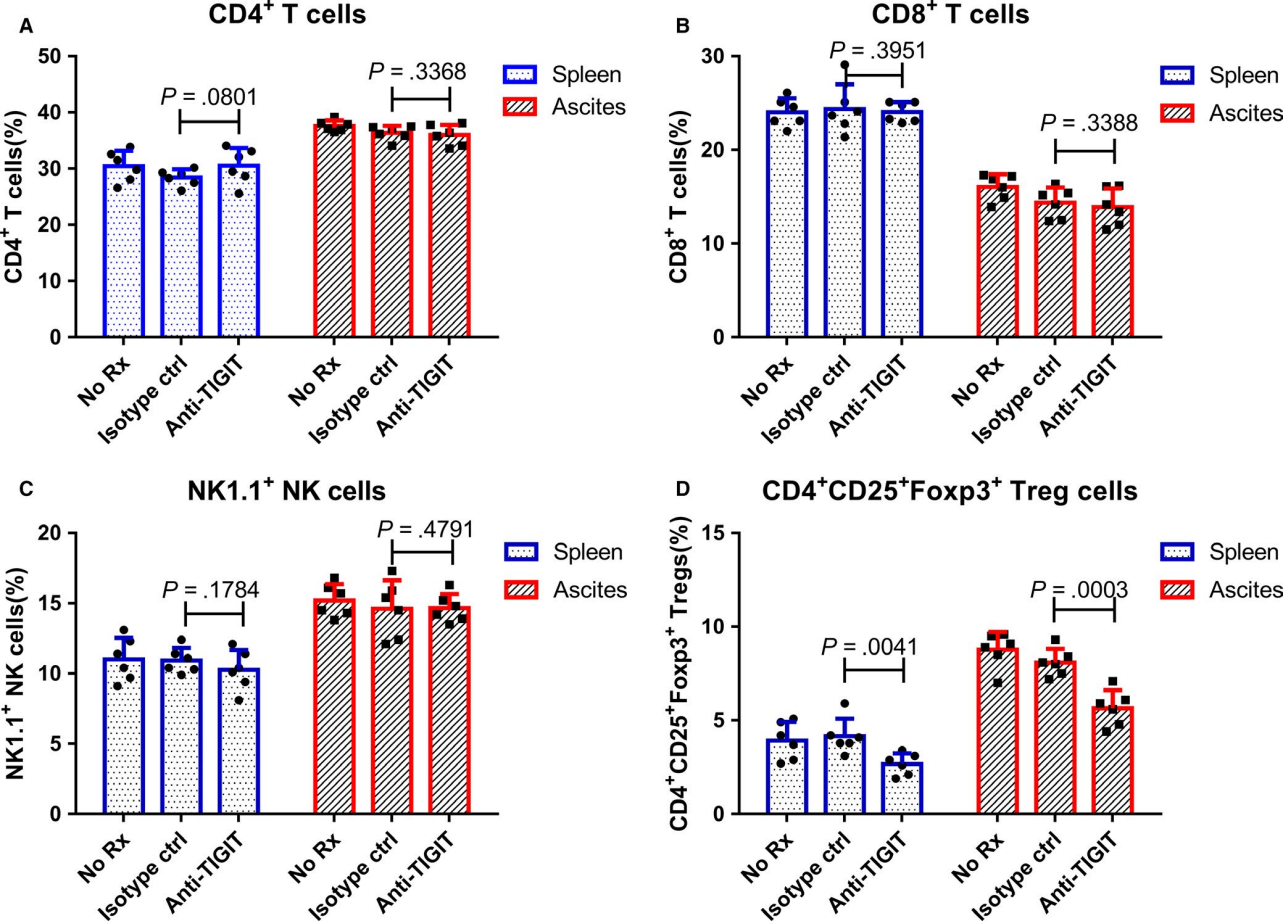
Anti‐TIGIT treatments reduced the proportion of CD4^+^ Tregs but had no effect on CD4^+^ and CD8^+^ T cells and NK cells. Mice (n = 6) were injected i.p. with 1 × 106 ID8 cells ten days before receiving treatments. OC mice were injected thrice in 4‐d intervals with 100 μg of isotype control or anti‐TIGIT mAb. Seven days after this, splenic and ascites cells were collected by Ficoll‐Paque density gradient centrifugation and were stained with specific antibodies; the proportions of CD4^+^ T cells (A), CD8^+^ T cells (B), NK cells (C), and CD4^+^ Tregs (D) were assessed by flow cytometry. Shown are the means ± standard deviation

To examine regulating functions of TIGIT on distinct immune cell types, ID8 cells were injected to establish a murine OC model. After 10 days, OC mice received a control isotype or anti‐TIGIT treatment. Seven days after this, lymphocytes of the spleen and ascites were collected, and the proportions of CD4^+^ T cells, CD8^+^ T cells, NK cells, and CD4^+^ Tregs were recorded using flow cytometry. Our results showed that anti‐TIGIT treatment reduced the proportions of CD4^+^CD25^+^Foxp3^+^ Tregs (spleen 2.67 ± 0.24% vs 4.15 ± 0.38%, *P* = .0041; ascites 5.65 ± 0.39% vs 8.08 ± 0.30%, *P* = .0003), compared to the control, whereas not effect on CD4^+^ (spleen 28.42 ± 0.60% vs 30.52 ± 1.30%, *P* = .0801; ascites 35.97 ± 0.76% vs 36.37 ± 0.53%, *P* = .3368), CD8^+^ T cells (spleen 24.05 ± 0.43% vs 24.37 ± 1.08%, *P* = .3951; ascites 13.90 ± 0.81% vs 14.35 ± 0.67%, *P* = .3388), and NK1.1^+^ NK cells (spleen 10.25 ± 0.58% vs 10.92 ± 0.37%, *P* = .1784; ascites 14.65 ± 0.41% vs 14.60 ± 0.83%, *P* = .4791; Figure 3) was observed.

### 3.4 Anti‐TIGIT treatment reduced immunosuppressive effects of CD4^+^ TREGS on CD4^+^CD25^−^ T effector cells

**Figure 4:**
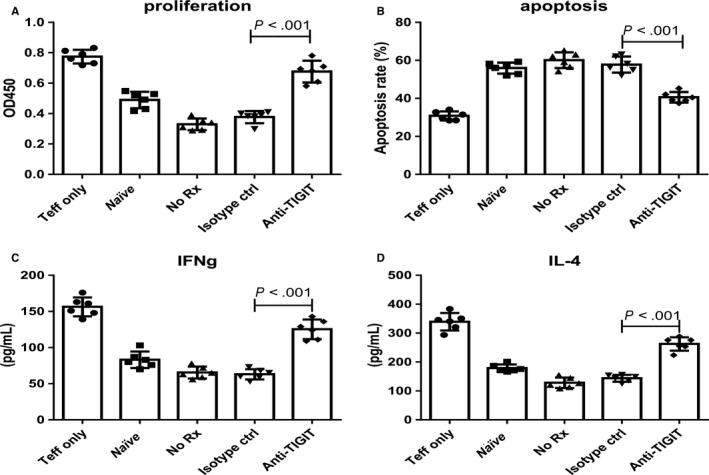
Anti‐TIGIT treatment reduced immunosuppressive functions of CD4^+^ Tregs. Mice (six individuals per group) were injected i.p. with 1 × 106 ID8 cells ten days before treatments. OC mice were injected thrice in 4‐d intervals with 100 μg of control or anti‐TIGIT mAb. Seven days after this, splenic CD4^+^ Tregs were isolated from different groups and were co‐cultured with normal CD4^+^ CD25^−^ T effector cells from normal mice in the stimulation of anti‐CD3 (5 μg/mL) and anti‐CD28 (2 μg/mL) in a ratio of 1:1 for 24 h. Proliferation (A), apoptotic rate (B), and secretive ability (IFN‐γ and IL‐4) (C and D) of CD4^+^ CD25^−^ T effector cells were determined. “Naïve” indicates normal splenic CD4^+^ Tregs co‐cultured with normal CD4^+^ CD25‐ T effector cells. Shown are the means ± standard deviation

To examine regulating functions of TIGIT on CD4^+^ Tregs, splenic CD4^+^CD25^+^Foxp3^+^ Tregs were isolated from naïve mice and different treatment groups of OC mice (no treatment, isotype control treatment, and anti‐TIGIT treatment). CD4^+^CD25^−^ T effector cells were isolated from naïve mice and were co‐cultured with different groups of isolated CD4^+^CD25^+^Foxp3^+^ Tregs. With inclusion of CD4^+^CD25^+^Foxp3^+^ Tregs derived from the anti‐TIGIT treatment group, proliferation of CD4^+^CD25^−^ T effector cells increased significantly (0.68 ± 0.07 vs 0.38 ± 0.04, *P* < .001), and apoptosis decreased (40.60 ± 2.77% vs 57.78 ± 4.23%, *P* < .001; Figure 4). The secretion function of CD4^+^CD25^−^ T effector cells as measured using IL‐4 (262.50 ± 23.08 pg/mL vs 143.80 ± 11.79 pg/mL, *P* < .001) and IFN‐γ (125.30 ± 13.52 pg/mL vs 63.00 ± 7.13 pg/mL, *P* < .001) was restored by the anti‐TIGIT treatment, compared to control (Figure 4).

### 3.5 Anti‐TIGIT treatment improved survival rates of OC mice

**Figure 5:**
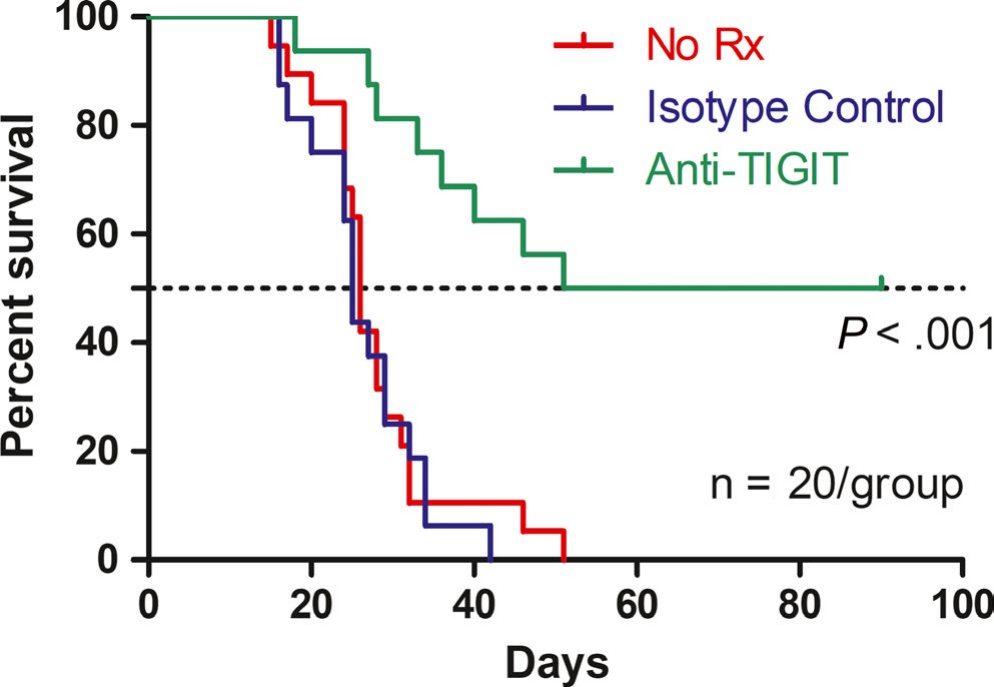
Anti‐TIGIT treatment significantly improved the survival rates of OC mice induced by ID8 cells. Mice (20 mice per group) were injected i.p. with 1 × 106 ID8 cells ten days before treatments. OC mice were treated thrice with 100 μg of control or anti‐TIGIT mAb in four‐day intervals. Survival was recorded daily, and mean survival time of mice with tumor growth was calculated. The survival rate was recorded, and a Kaplan‐Meier survival curve was produced

Mice (n = 20) injected i.p. with 1 × 10^6^ ID8 cells 10 days before were injected thrice in 4‐day intervals with 100 μg of control medium or anti‐TIGIT mAb. Survival rates were recorded and a Kaplan‐Meier survival curve was produced. The survival rate of the anti‐TIGIT treatment group was significantly higher than that of the isotype control and of the no‐treatment group (Figure 5).

## 4 DISCUSSION

Immune dysfunction induced by infiltrated Tregs is strongly associated with mortality in OC patients. TIGIT, a recently identified co‐inhibitory receptor expressed on the surface of various of lymphoid cells, predominantly regulates immune responses through Tregs. Our results showed that expression of TIGIT increased in immune cells of a murine OC model, particularly in Tregs. Inhibition of TIGIT signaling improved survival of OC mice, predominantly through inhibiting Tregs immune function. We conclude that TIGIT enhanced Tregs responses and mediated immune suppression in OC. Furthermore, this strongly suggests that TIGIT affects immune responses during OC under participation of CD4^+^ Tregs. TIGIT signaling thus contributes to immunosuppression during OC through CD4^+^ Tregs.

CD4^+^ Tregs are a group of specialized immune cells that play a crucial role for immune homeostasis.^18^ Several studies have confirmed that peripheral Tregs abundance is associated with poor prognoses in OC cases, and depleting Tregs during OC will improve immunity and may exert therapeutic effects.^15^, ^19^, ^20^ However, the mechanisms by which Tregs inhibit antitumor immune responses are not comprehensively understood. Our study revealed one possible mechanism affecting Tregs: the TIGIT signal pathway.

Our results confirmed that during OC, TIGIT predominantly regulates immune responses via Tregs. The TIGIT signaling pathway also affects immune responses through CD4^+^ and CD8^+^ T cells in cases of systemic lupus erythematosus, rheumatoid arthritis,^21^, ^22^ and human gastric cancer.^23^ A study on MC38 colon carcinoma revealed that 1B4 anti‐TIGIT antibody exerts deficient antitumor activity,^16^ which seems to contradict our conclusions. However, expression of TIGIT known to be substantially higher during OC than in cases of colon carcinoma.^24^, ^25^ Moreover, expression levels of TIGIT have been shown to differ between tumor sites,^24^ which may in part explain the observations made in the present study.

Our study revealed that TIGIT antibody treatment not only reduced the frequency of Tregs, but also decreased suppressive capacity of splenic Tregs. The underlying mechanisms, however, are considerably complicated. First, TIGIT expression is strongly correlated with stability of Tregs in humans,^26^ which may contribute to the reduced abundance of Tregs. Second, TIGIT signaling drives a cell‐intrinsic gene program in Tregs that may be a cause of decreased suppressive capacity.^14^ Third, low production of IL‐10 after TIGIT antibody treatments may also contribute to decreased suppressive capacity. Our study demonstrated that TIGIT was involved in regulating Tregs during OC, and our results suggest new directions for future research on Tregs functioning in OC cases.

The present study, however, has several limitations. First, we did not assess the development of immune memory in tumor‐bearing mice treated with anti‐TIGIT. Second, recent research showed that the Fc region of mAbs targeting TIGIT may enhance antigen‐specific T‐cell responses and tumoricidal activity.^27^ The specific anti‐TIGIT antibodies (Clone 1B4) used in this study may also have impacted our experimental results. Third, we only assessed immune responses of CD4^+^ Tregs and did not evaluate the overall immune status in vivo. Further studies are needed to examine potential confounding effects in order to comprehensively explain the mechanisms underlying the effects of TIGIT.

## 5 CONCLUSIONS

We report that TIGIT enhanced the immune response of Tregs and mediated immune suppression in a murine OC model. Mediators that targeting TIGIT and Tregs may thus be novel therapeutic targets for the treatment of OC.

## CONFLICTS OF INTEREST

The authors declare no competing interests.

## AUTHOR CONTRIBUTIONS

FC conceived the project and wrote the manuscript. YX and YC performed experiments and analyzed the data. SS contributed reagents/materials/analysis tools. All authors discussed the results and reviewed the manuscript.

## ETHICS APPROVAL

All experimental protocols utilizing animals were conducted with approval by the Tianjin Medical University's ethics committee.

## Data Availability

Data produced in this study will be made available by the corresponding author upon request.
